# CD10 marks non-canonical PPARγ-independent adipocyte maturation and browning potential of adipose-derived stem cells

**DOI:** 10.1186/s13287-021-02179-y

**Published:** 2021-02-04

**Authors:** Smarajit Chakraborty, Wee Kiat Ong, Winifred W. Y. Yau, Zhihong Zhou, K. N. Bhanu Prakash, Sue-Anne Toh, Weiping Han, Paul M. Yen, Shigeki Sugii

**Affiliations:** 1 Fat Metabolism and Stem Cell Group, Singapore Bioimaging Consortium (SBIC), Agency for Science, Technology and Research (A*STAR) Singapore, 11 Biopolis Way, Singapore, 138667 Singapore; 2 Xenobiology Division, Institute of Bioengineering and Nanotechnology (IBN) Singapore, A*STAR, 31 Biopolis Way, Singapore, 138669 Singapore; 3 School of Pharmacy, Monash University Malaysia, 47500 Selangor, Malaysia; 4 Cardiovascular and Metabolic Disorders Program, Duke-NUS Medical School Singapore, 8 College Road, Singapore, 169857 Singapore; 5 Signal and Image Processing Group, SBIC, A*STAR Singapore, 11 Biopolis Way, Singapore, 138667 Singapore; 6 Department of Medicine, Yong Loo Lin School of Medicine, National University of Singapore, 5 Lower Kent Ridge Road, Singapore, 119074 Singapore; 7 Laboratory of Metabolic Medicine, SBIC, A*STAR Singapore, 11 Biopolis Way, Singapore, 138667 Singapore

**Keywords:** Biomarker, adipogenesis, Quality, adipose-derived mesenchymal stem cells, Adipocytes, beige, Adipocytes, non-canonical activation, Lipolytic pathways, Seahorse analysis, oxidative metabolism, Neprilysin, NEP, MME, Drug screening, nuclear receptor superfamily, Lipid droplets accumulation

## Abstract

**Background:**

Effective stem cell therapy is dependent on the stem cell quality that is determined by their differentiation potential, impairment of which leads to poor engraftment and survival into the target cells. However, limitations in our understanding and the lack of reliable markers that can predict their maturation efficacies have hindered the development of stem cells as an effective therapeutic strategy. Our previous study identified CD10, a pro-adipogenic, depot-specific prospective cell surface marker of human adipose-derived stem cells (ASCs). Here, we aim to determine if CD10 can be used as a prospective marker to predict mature adipocyte quality and play a direct role in adipocyte maturation.

**Methods:**

We first generated 14 primary human subject-derived ASCs and stable immortalized CD10 knockdown and overexpression lines for 4 subjects by the lentiviral transduction system. To evaluate the role of CD10 in adipogenesis, the adipogenic potential of the human subject samples were scored against their respective *CD10* transcript levels. Assessment of *UCP1* expression levels was performed to correlate CD10 levels to the browning potential of mature ASCs. Quantitative polymerase chain reaction (qPCR) and Western blot analysis were performed to determine CD10-dependent regulation of various targets. Seahorse analysis of oxidative metabolism and lipolysis assay were studied. Lastly, as a proof-of-concept study, we used CD10 as a prospective marker for screening nuclear receptor ligands library.

**Results:**

We identified intrinsic CD10 levels as a positive determinant of adipocyte maturation as well as browning potential of ASCs. Interestingly, CD10 regulates ASC’s adipogenic maturation non-canonically by modulating endogenous lipolysis without affecting the classical peroxisome proliferator-activated receptor gamma (PPARγ)-dependent adipogenic pathways. Furthermore, our CD10-mediated screening analysis identified dexamethasone and retinoic acid as stimulator and inhibitor of adipogenesis, respectively, indicating CD10 as a useful biomarker for pro-adipogenic drug screening.

**Conclusion:**

Overall, we establish CD10 as a functionally relevant ASC biomarker, which may be a prerequisite to identify high-quality cell populations for improving metabolic diseases.

**Supplementary Information:**

The online version contains supplementary material available at 10.1186/s13287-021-02179-y.

## Background

White adipose tissue (WAT) is primarily involved in storing lipids and secrete bioactive adipokine and cytokine dysfunction of which leads to reduced adipogenesis, metabolic syndrome, inflammation, insulin resistance, and type 2 diabetes [1–5]. Adipose-derived stem/stromal cells (ASCs) which constitute ~ 1% of WAT are well defined as a mesenchymal stem cell type by the International Society for Cell and Gene Therapy (ISCT) based on their adherence, multipotency, and presence of selected markers [6]. By virtue of their differentiative and immunobiological properties, ASCs promise to be immunomodulators and gene delivery vehicles and possess a significant therapeutic impact in the field of regenerative medicine [7–9]. Several lines of evidence have shown pathophysiological differences between subcutaneous fat versus visceral fat in terms of interaction, secretion, insulin sensitivity, inflammation, circulation, maturation, metabolic, and lipid profiles [10–12]. Whether these differences originate from the stem cell populations only recently have started to be revealed.

Our group previously presented definitive cell surface markers and molecular signatures of ASCs derived from subcutaneous fat and visceral fat depots [6, 13]. Our high content screening assay of over 240 human cell surface markers identified CD10 (a.k.a. membrane metalloendopeptidase, MME) to be a subcutaneous fat-specific factor. We further showed that these markers can be used to enrich distinct populations of stem cells by their potential adipogenic capabilities; when sorted, CD10^hi^ ASCs differentiated into adipocytes better than CD10^lo^ counterparts [6]. These results implicated potential use of CD10 as a prospective marker for high-quality adipogenesis and its potential direct role in the adipogenic process. However, it remained to be established if this stem cell marker can be used to indicate later phase adipocyte maturation capacities of ASCs across different human subject samples and if CD10 plays a direct role in terminal differentiation.

In contrast to WAT, brown adipose tissue (BAT) specializes in expending energy and generating heat through the specific function of uncoupling protein-1 (UCP1), which in turn leads to improved systemic glucose and lipid homeostasis [14, 15]. Although BAT is abundant in rodents and infants but not in human adults, inducible, browning (also termed “beige” or “brite”) adipocytes are discovered present inside subcutaneous WAT and resemble biological characteristics of BAT [16–18]. Thus, inducible characteristics of beige cells make them a promising therapeutic target for obesity treatment. There is no report of stem cell markers to our knowledge that indicate beiging/browning abilities of ASCs.

In this manuscript, we aim to determine if CD10 can be used as a marker to predict adipocyte maturation and browning potential of ASCs. We successfully delineate the impact of CD10 on adipogenic differentiation capacity by using human knockdown (KD) and overexpression (OE) lines. We report that CD10-driven adipocyte maturation of ASCs is independent of the classical PPARγ-dependent adipogenic pathway and thus, is non-canonical. Further, we investigate if CD10 can be used as a biomarker to screen for ASCs with higher adipogenic capacities, which is a prerequisite to improve quality of fat cells important for preventing metabolic abnormalities.

## Materials and methods

### Isolation and culture of ASCs

WAT was isolated from the subcutaneous (abdominal region) depot from all the human volunteers undergoing bariatric surgery with approval from the Domain Specific Review Board at National Healthcare Group, Singapore. Information of the 12 donors is described in Supplementary Table S1. ASCs were isolated and enriched by serial passage culture of stromal vascular fractions (SVF), as described previously [6, 19]. Immortalized ASCs were established from subject S29, with lentivirus using the pLVX-puro-Myc plasmid and Lenti-X HT Packaging System. The immortalized ASCs retained normal differentiation levels up to passage 30 (data not shown).

### Adipogenesis, browning, and AdipoRed staining

Two days after reaching confluency, ASCs were induced with adipogenic cocktail containing 1 mM dexamethasone (Sigma, USA), 0.5 mM IBMX (Sigma, USA), and 167 nM insulin plus (Gibco, USA) 100 mM indomethacin (Sigma, USA) as reported previously [6]. On D6, cells were switched to medium with 167 nM insulin and 1 mM dexamethasone and maintained until D12. The medium was changed every 3 days till D12. To induce browning, differentiated ASCs were treated with either 10 mM Forskolin (FSK) (Sigma, USA) or DMSO control for 6 h. For the AdipoRed staining of lipid droplets (LDs), AdipoRed™ Assay Reagent (Lonza, USA) was used in a 96-well format according to the manufacturer’s instructions with modifications. The cells were stained with the dye reagent for 30 min followed by 2X wash with HBSS. The nuclei of cells were then counterstained with Hoechst 33342 (Thermo Fisher Scientific, USA) for 15 min before proceeding for imaging.

### Oil Red O staining

After adipogenesis, the cells were fixed in 3.7% formaldehyde in PBS for 1 h, washed with 60% isopropanol, air-dried, and stained with Oil Red O solution (in 60% isopropanol) as described previously [6]. Staining was extracted by isopropanol and its absorbance was measured at 500 nm by a spectrophotometer.

### Microscopy

Imaging was performed by using a high-content automated fluorescence microscope system ImageXpress (Molecular Devices, USA) equipped with AgileOptix™ technology. Images were obtained with a × 10 objective lens in a 100w pillar diascopic illuminator with TE-C ELWD condenser. Software ImageJ was used to analyze the images. Two sets of images were taken corresponding to (i) AdipoRed™ reagent emission wavelength (536/40 nm) upon 506 nm donor excitation and (ii) Hoechst 33342 emission wavelength (447/60 nm) upon 409 nm excitation.

### Quantification of lipid droplets

A comprehensive automated MATLAB algorithm, which was adapted from our previously developed computational method [20], was used to count the nuclei number, measure the number of LDs normalized to the nuclei counts, and score the percentage of nuclei associated with lipids. Intensity, neighborhood, and shape features were used in the method to characterize the LDs and their association to nuclei that are used in the calculation of the ratio of total lipid intensity (total amount in pixels) to the corresponding nuclei intensity (total amount in pixels).

### Knockdown and overexpression of CD10

Short hairpin RNA (shRNA) constructs were annealed, phosphorylated, ligated, and cloned into lentiviral pKAMU H1 vector backbone, using BamHI/EcoRI restriction sites. The shRNA construct was then transformed into *Escherichia coli* DH5α cells*.* Positive clones were selected by sequencing the plasmid vector. Lentivirus was produced by co-transfecting human embryonic kidney 293 (HEK293T) cells with pKAMU containing either scrambled sequence (control) or CD10 shRNA, along with pMDL, pREV, and pVSVG. Media containing virus was collected after 48 h, which was subsequently concentrated by ultracentrifugation. Immortalized ASCs were then infected with either control or CD10-shRNA lentiviral vectors at a multiplicity of infection (MOI) 10. ASCs were incubated with puromycin for 48 h post-infection and kept for at least a week to select for successfully infected ASCs.

To study overexpression of CD10, the control vector (iRFP720-P2A) was constructed by cloning P2A, a porcine teschovirus-1-derived self-cleaving 2A peptide, tandemly with image tracking reporter iRPF720 by 2A technology [21]. The iRFP720 gene was amplified from piRFP720-N1 (Addgene plasmid #45461) with a flanking 5 prime NheI site and 3 prime NotI sites including the full P2A sequence. The purified iRFP720 gene was then cloned into the multiple cloning sites (MCS) of expression lentivector pCDH-EF1-MCS-pA-PGK-copGFP-T2A-Puro (Addgene plasmid #CD550A-1). The overexpression vector (iRFP720-P2A-CD10) was designed in 2 steps. First, the CD10 gene was PCR amplified flanked by 5 prime NheI-SalI site and 3 prime Not I site and cloned into the MCS of vector pCDH-EF1-MCS-pA-PGK-copGFP-T2A-Puro. Next, the iRFP720-P2A fragment containing the 5 prime NheI site and 3 prime SalI site was PCR amplified and cloned into the respective NheI-SalI site of the vector obtained from the first step. All the above-mentioned vectors were verified by further sequencing of the identified clones. These lentiviral vectors were infected to immortalized ASCs to establish stable lines.

### Quantitative real-time PCR

Total RNA from the cultured cells was extracted using TRIzol reagent (Invitrogen, USA) and treated with DNase I to remove genomic DNA. cDNA conversion and analysis was done as previously described [6]. Relative mRNA levels were calculated and normalized to that of human ribosomal protein L27 (*RPL27*). The primers are listed in Supplementary Table S2.

### Seahorse XF analyzer measurement for mitochondrial oxygen consumption rate (OCR)

ASCs were seeded on XF-24-well culture microplates and allowed to differentiate as mentioned above. Differentiated ASCs were then treated with either 10 mM Forskolin (FSK) or DMSO control for 6 h. After treatment, oxygen consumption was measured using a microplate (type XF24) extracellular analyzer (Seahorse Bioscience, Billerica, MA, USA) as described in [22].

### Immunoblot analysis

Cells were dissociated in RIPA buffer (50 mM Tris-HCl, pH 8.0, 150 mM sodium chloride, 1% Triton X-100 (Bio-Rad), 0.5% sodium deoxycholate (Sigma, USA), 2 mM EGTA, 2 mM EDTA, 0.1% sodium dodecyl sulfate, protease inhibitors (Sigma, USA), and phosphatase inhibitors (Sigma, USA); proteins were denatured by boiling in Laemmli sample buffer (250 mM Tris-HCl, pH 7.4, 2% v:v sodium dodecyl sulfate, 25% v:v glycerol, 50 mM DTT, 0.01% w:v bromophenol blue). Equal amount of proteins was resolved on sodium dodecyl sulfate-nefapolyvinylidene difluoride membranes (Bio-Rad) using the TransBlot® Turbo™ Transfer System (Bio-Rad). The following antibodies were used to detect the target proteins. CD10 (Leica Microsystmes), PPARG, phospho-AKT (Ser473), AKT, phospho-p70S6 (Thr389), phospho-70S6 (Thr470), phospho-AMPK (P-T172), AMPK, p62, LC3, ATF4, ATF6, phospho-HSL (Ser660), HSL, phosphor-ATGL (Ser406), ATGL, and COXIV. HRP-conjugated IgG (Santa Cruz) was used as the secondary antibody. The membranes were stained with Pierce™ ECL Plus (Thermo Fischer Scientific, USA) luminol/*enhancer* reagent and the X-ray film was developed by autoradiography. Antibodies used in this study are listed in Supplementary Table S3.

### Determination of lipolysis in matured ASCs

Lipolysis was determined by estimating triglyceride breakdown and release of glycerol and non-esterified fatty acids (NEFA) from matured ASCs. Intracellular triglyceride levels were measured of fully differentiated ASCs by the Triglyceride Colorimetric Assay Kit (Caymen Chemical, USA). The amount of NEFA present in the pellet as well as released outside were estimated by the Free Fatty Acid Fluorometric Assay Kit (Caymen Chemical, USA). Glycerol release was measured by Glycerol Colorimetric Assay Kit (Caymen Chemical, USA). All the results were normalized per milligram of protein present.

### Nuclear ligand screening

Screening was carried out using Screen-Well nuclear receptor ligand library (Enzo BML-2802) with 76 synthetic ligands (10 μM) of nuclear receptors (AR, CAR, ER, GR, FXR, LXR, PPAR, PXR, RAR, RXR, VDR). The assays were performed by immunofluorescence study using ImageXpress high content screening system and flow cytometry analysis with BD LSR Fortessa on ASCs from S13 that had been incubated with 10 μM of the respective ligands for ~ 68 h. The screening was done by using high-throughput flow cytometry and image-based high content analyses (Supplementary Table S4). Selected candidate ligands of interest from the first screening were further tested by individual flow cytometry analysis (Fig. S4). Positive hits were applied on ASCs from S23 and verified by flow cytometry analysis.

### Flow cytometry

ASCs were trypsinized and a total of 2 × 10^5^ cells were suspended in 0.2 ml staining buffer (DMEM without phenol red with 2% FBS) (Thermo Fisher Scientific, USA) for immunostaining. The cells were incubated for 30 min on ice, with a FITC-conjugated antibody against CD10 (BD Biosciences, USA). The stained cells were washed and suspended in sorting buffer (PBS with 0.5% BSA and 2 mM EDTA) before analysis by flow cytometry (LSR FORTESSA, BD Biosciences).

### Statistical analysis

Results were represented as means ± SEM. Student’s *t* test was used to determine differences in means between two groups. The *p* value was calculated using ANOVA for multiple comparisons with corrections and *p* < 0.05 being considered as significant. Correlation coefficient (*R*) value was calculated from the eq. [*R*^*2*^ = \frac{\sum{(x-m_x)(y-m_y)}}{\sqrt{\sum{(x-m_x)^2}\sum{(y-m_y)^2}}}; m, mean; x, relative *CD10* level; y = relative LDs level].

## Results

### Intrinsic levels of CD10 determine the adipogenic maturation potential of ASCs

**Fig. 1:**
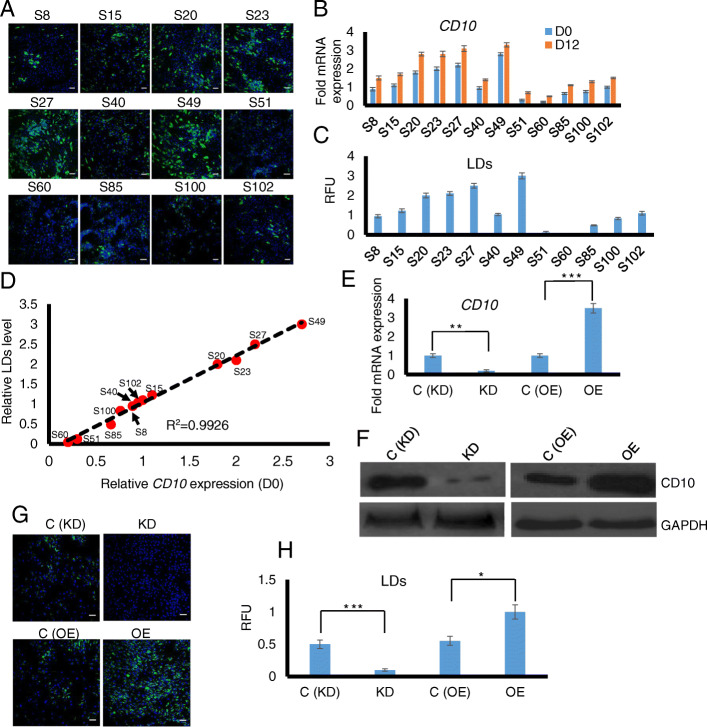
Intrinsic levels of CD10 positively correspond to lipid accumulation. **a** Representative merged images of AdipoRed staining of lipids (in green) and Hoechst 33342 staining of nuclei (in blue) across differentiated ASCs from 12 subjects (between passage 6 and 8) are shown. The scale bar represents 100 μm. **b** *CD10* levels at D0 (basal undifferentiated) and at D12 (differentiated) of ASCs from the 12 subjects are shown based on qRT-PCR analysis. Fold expression changes of *CD10* are compared to the S8. Each value is the mean ± SEM from three independent wells for qRT-PCR of each population performed in triplicates. **c** The LDs are quantified in the differentiated ASCs of the 12 subjects by using a MATLAB algorithm as a read-out for adipogenesis levels. Each value is the mean ± SEM (*n*=6). **d** Correlation of *CD10* levels at D0 and lipids at D12 post-differentiation are shown. *CD10* levels are highly correlated with the lipids accumulated upon differentiation (Correlation coefficient *R* = 0.9962). **e** The relative mRNA expression levels of *CD10* by qRT-PCR in CD10 knockdown (KD), CD10 overexpression (OE) compared with their respective controls C (KD) and C (OE) ASCs are shown normalized to *RPL27*. Fold expression changes are compared to the C (KD) level. Each value is the mean ± SEM from three independent replicates. **f** Western blot analysis shows the protein expression of CD10 in CD10 KD and CD10 OE ASCs compared to the respective controls. GAPDH was used as the loading control. **g** Representative merged images of AdipoRed staining of lipids (in green) and Hoechst 33342 staining of nuclei (in blue) of CD10 KD, CD10 OE and the control ASCs lines are shown. The scale bar represents 100 μm. **h** Quantification of the lipid content was done by using a MATLAB algorithm. CD10 OE and CD10 KD ASC populations showed respective increase and decrease in lipids compared to the controls. Each value is the mean ± SEM for each population performed in five-independent batches. All statistical analysis was performed using Student’s paired *t* test, ***p < 0.001, **p < 0.01, *p < 0.05

Based on our previous finding, we postulated that intrinsic levels of CD10 might positively predict the potential of ASC maturation into adipocytes [6]. We investigated human subjects-derived ASCs isolated from the subcutaneous depot from 12 donors within low passages to avoid possible cellular variation. These ASCs were subjected to the standard in vitro adipogenic cocktail and accumulation of LDs was quantified. To evaluate expression changes of *CD10*, quantitative real-time PCR (qRT-PCR) was performed in non-differentiated (D0; basal) and mature (D12) ASC populations. ASCs with higher basal *CD10* levels (S49) showed robust adipogenesis whereas the lowest basal *CD10* expressing ASC (S60) accumulated minimum LDs as quantified by the AdipoRed assay (Fig. 1a–c). All the 12 subjects showed moderate increase in *CD10* expression from D0 to D12 during the course of differentiation (Fig. 1b), in agreement to our previous report [6]. The CD10 levels at D12 are consistent with those at D0 relative to individual subjects. Near-complete correlation (Pearson’s *R*^2^ = 0.9926) was observed between the basal *CD10* levels (at D0) and LDs accumulation (at D12), indicating the intrinsic CD10 level as a strong predictor of mature adipocyte quality (Fig. 1d). Overall, our data highlights functional heterogeneity within subject-derived ASCs isolated from the same subcutaneous fat depot and suggests that high baseline CD10 is predictive of good adipogenic potentials in ASCs.

### CD10 directly impacts adipocyte maturation of ASCs

To study the functional relevance of CD10 in regulating adipogenic maturation, we created stable knockdown (KD) and overexpression (OE) lines. Immortalized ASCs were stably transduced with CD10 shRNA or empty vector (control) to obtain CD10 KD and control cell populations. On the other hand, ASCs were transduced with CD10 overexpression or vector control as described in the “Materials and methods” section to obtain the CD10 OE and control cell lines. To examine the extent of overexpression and knockdown by the lentiviral transduction system, we investigated the expression of CD10 at both RNA and protein levels. The qRT-PCR analysis showed about 5-fold decrease in *CD10* expression in the KD cells and 3.5-fold increase in OE cells compared to the respective controls (Fig. 1e). Changes in the protein expression were also confirmed by Western blot by using CD10-specific antibody (Fig. 1f). Next, we subjected the ASC lines into an in vitro standard adipogenic differentiation cocktail as described in the “Materials and methods” section. AdipoRed, a specific fluorescence dye for neutral lipids, was used to stain the LDs, and quantification was done by an automated algorithm based on the previously developed protocol (see the “Materials and methods” section) [20]. KD of CD10 significantly reduced LD accumulation upon differentiation, while CD10 OE enhanced LD signals, suggesting a positive correlation between basal CD10 levels and adipocyte maturation (Fig. 1g, h). In addition, we generated knockdown cell lines of 3 other subjects to further validate CD10-dependent adipocyte maturation by using a different LD-specific dye, Oil Red O. As expected, knockdown cells expressing low *CD10* levels as determined from the qRT-PCR analysis (Fig. S1A – S1C) exhibited significantly reduced LDs compared to the controls (Fig. S1D and Fig. S1E). Collectively, we conclude that CD10 directly and positively regulates adipocyte maturation of human ASCs.

### CD10-rich ASCs show increased beige phenotype upon stimulation

**Fig. 2:**
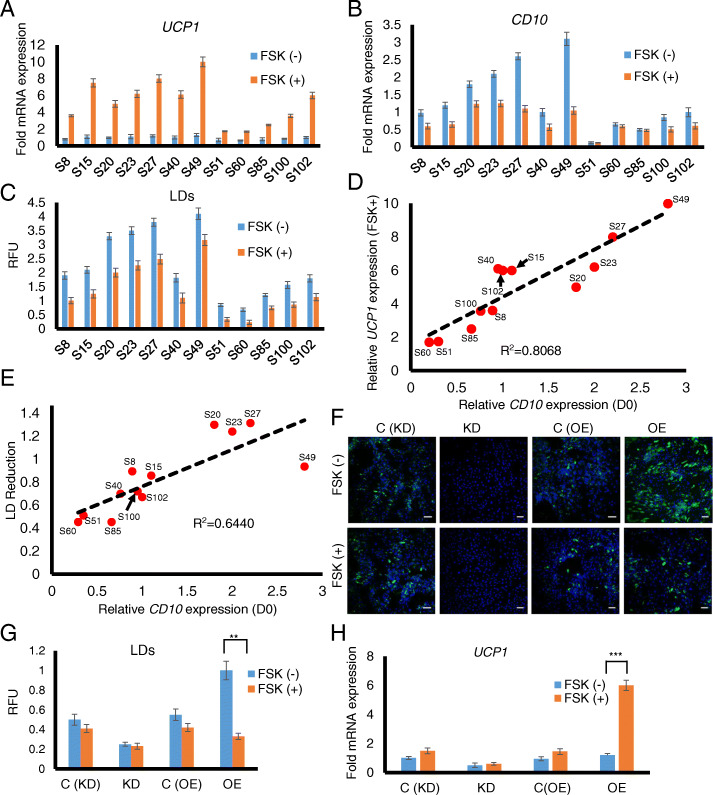
High *CD10* expressing ASCs show increased browning capacity. **a** Relative mRNA levels of *UCP1* are quantified before and after FSK treatment by qRT-PCR normalized to *RPL27*. Fold expression changes are compared to the subject S8 without FSK treatment. Each value is the mean ± SEM from three independent replicates. **b** Relative mRNA levels of *CD10* are quantified at D0 and D12 by qRT-PCR normalized to *RPL27*. Fold expression changes are compared to the subject S102 (D0). Each value is the mean ± SEM from three independent replicates. **c** The LDs are quantified by using MATLAB analysis before and after FSK treatment. **d** Correlation of *CD10* levels with *UCP1* expression are shown with the Pearson coefficient *R*^2^ value. Good correlation is observed between *UCP1* induction and *CD10* levels (*R* = 0.8610). **e** Correlation between intrinsic *CD10* levels with reduction in LDs following browning are shown with the Pearson coefficient *R*^2^ value. **f** Representative merged images of AdipoRed staining of lipids (in green) and Hoechst 33342 staining of nuclei (in blue) in CD10 knockdown (KD) and overexpression (OE) cells with controls with (out) FSK treatment. The scale bar represents 100 μm. **g** The LDs are quantified by using MATLAB analysis as described in Experimental Procedures. The values are normalized to CD10 OE ASCs. Each value is the mean ± SEM from three independent replicates. **h** Relative mRNA levels of browning marker gene UCP1 are quantified by qRT-PCR normalized to RPL27. Fold expression changes are compared to CD10 OE cells before FSK treatment. Each value is the mean ± SEM from three independent replicates. All statistical significance was assessed by using Student’s paired t-test: ***p < 0.001, **p < 0.01, *p < 0.05

We next tested if CD10 levels also predict the browning potential of mature ASCs. ASCs were differentiated with the standard adipogenic differentiation cocktail for 12 days and then treated with Forskolin (FSK) for 6 h to induce browning. Browning assessment from *UCP1* expression and adipogenesis from LDs quantification were performed before and after the FSK treatment. Induced browning as evidenced by *UCP1* induction resulted in decreased lipid accumulation as quantified from the AdipoRed staining of LDs (Fig. 2a–c). Importantly, cells with higher intrinsic CD10 levels showed increased *UCP1* upregulation compared to those with low CD10 levels (Fig. 2a, b). *UPC1* upregulation showed good correlation to intrinsic CD10 levels (Pearson’s *R*^2^ = 0.8068) (Fig. 2d). Next, we compared if the reduction in LD accumulation as a consequence of induced browning is related to intrinsic CD10 levels. We observed moderate correlation (Pearson’s *R*^2^ = 0.6440) between lipid reduction and CD10 levels indicating CD10 as a potential marker of browning potential of ASCs (Fig. 2e). To investigate a direct effect of CD10 on browning potential of ASCs, we next compared *UCP1* expression of CD10 OE and KD ASCs. As expected, CD10 OE ASCs showed the highest *UCP1* induction and maximum reduction in LDs upon browning treatment, whereas CD10 KD ASCs showed no *UCP1* induction and no LDs reduction (Fig. 2f–h). Overall, our results establish CD10 as an additional functional determinant of browning potential of ASCs.

### CD10-induced adipocyte maturation of ASCs is non-canonical

To elucidate the function of CD10, we first measured the expression levels of key adipogenic transcriptional factor, peroxisome proliferator-activated receptor gamma (*PPARG*), and specific late adipogenic gene, Fatty acid-binding protein 4 (*FABP4*; a.k.a. aP2) by qRT-PCR. Interestingly, *PPARG* and *FABP4* levels across all 12 subjects remain unchanged as also observed in the differentiated CD10 KD or CD10 OE lines, which did not show significant changes compared to the respective controls (Fig. S2A – S2C). These results indicate that CD10-induced lipid accumulation is not via the classical adipogenic pathway mediated by the master regulator PPARγ. We next investigated whether CD10 regulates other signaling pathways involved in adipocyte differentiation by examining the activation of specific targets such as AKT (PI3K/Akt pathway), p70S6 (mTOR pathway), 5′ AMP-activated protein kinase (AMPK; AMPK pathway), p62 and LC3 (autophagy pathway), and Activating Transcription Factor 4 and 6 (ATF4 and ATF6; endoplasmic reticulum stress pathway). None of their players exhibited significant changes in their expression of both total protein and phosphorylation levels as evidenced by Western blot analysis (Fig. S2D), suggesting that CD10 acts independent of any of these pathways. These results implicate a more direct role of CD10 in regulating lipid formation of mature ASCs without going through the canonical adipogenic pathways.

### CD10 promotes lipid accumulation of mature ASCs by inhibiting lipolysis

**Fig. 3:**
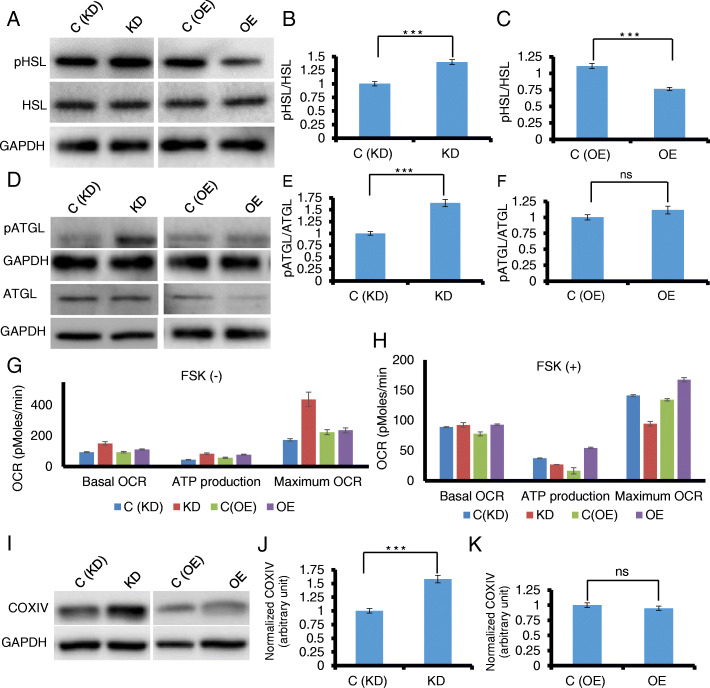
CD10 inhibits endogenous lipolysis resulting in higher lipid accumulation in mature ASCs. Western blot analysis shows detection of **a** phosphorylated form of HSL and **d** ATGL using antibodies against phospho-HSL (P-S660), total HSL, phospho-ATGL (P-S406), and total ATGL in CD10 KD, OE, and control cells. Quantification of HSL phosphorylation are shown in **b** CD10 KD and **c** CD10 OE cells. Similar phosphorylation levels are shown for ATGL in **e** CD10 KD and **f** CD10 OE cells. CD10-mediated alteration on mitochondrial function expressed as Oxygen consumption rate (OCR; in pMoles/min) are shown in mature ASCs **g** before or **h** after FSK treatment. Western blot analysis showing the **i** expression of COXIV and its relative quantification between **j** CD10 KD and **k** OE cells compared to the controls. For all the blots, GAPDH was used as internal control, and statistical analysis was performed using Student’s paired *t* test (*n* = 3), ***p < 0.001, *p < 0.05, ns denotes non-significant, *p* > 0.05

We reasoned that CD10 may be directly involved in de novo lipogenesis or lipolysis to regulate lipid accumulation in differentiated ASCs. We did not observe any significant changes in the expression level of fatty acid synthase (FAS) or phosphorylation of acetyl-CoA Carboxylase (ACC) in the CD10 KD or CD10 OE lines compared to the controls, suggesting non-involvement of CD10 in de novo lipogenesis (data not shown). Next, we investigated if CD10 regulates lipolysis by measuring the activation of key enzymes involved in intracellular lipid catabolism including hormone-sensitive lipase (HSL) and adipose triglyceride lipase (ATGL) of differentiated ASCs. Compared to the control cells, CD10 KD cells showed a significant increase in the phosphorylation of both HSL and ATGL, whereas CD10 OE cells exhibited reduced activation (Fig. 3a–f). Our data suggest that CD10 mediates lipid accumulation by suppressing the activation of HSL and ATGL during adipogenic differentiation of ASCs. Next, we postulated whether ATGL and HSL activation in mature CD10 KD cells results in heightened mitochondrial respiration/β-oxidation, which is caused by excessive breakdown of triglycerides (TGs) from LDs. Indeed, the Seahorse XF Cell Mito Stress assay recorded increased basal respiration, ATP production, and further enhanced maximal mitochondrial respiration, accounting for the result of higher endogenous lipolysis in mature CD10 KD cells (Fig. 3g). Increased expression of Cytochrome c Oxidase Subunit IV (COXIV), an essential protein for mitochondrial respiration, further validates heightened mitochondrial activity of CD10 KD cells (Fig. 3i–k). Next, we set out to quantify CD10-regulated intracellular lipolysis by comparing the amount of TGs accumulation and resultant release of free fatty acids (FAs) and glycerol between CD10 KD, OE, and control cells. In agreement with our finding, CD10 OE cells showed the highest accumulation of TGs and minimal release of FAs and glycerol into the media (Fig. S3). CD10 KD cells displayed lower triglyceride accumulation and maximum release of FAs and glycerol, conforming accelerated lipolysis (Fig. S3). Taken together, CD10 exerts a direct role in lipid accumulation during adipogenic maturation of ASCs by regulating intracellular lipolysis. As we observed that stem cell CD10 levels also positively mark the browning potential of ASCs (Fig. S1I), we reasoned that increased adipocyte browning may correspond to higher mitochondrial function in CD10 OE cells. Basal and maximum mitochondrial respiration rates along with ATP production were significantly raised in CD10 OE cells after induction with FSK, validating a strong association between adipocyte browning and mitochondrial activity (Fig. 3h). Collectively, while CD10 directly regulates lipolysis and mitochondrial metabolism, it also impacts the ability of mature ASCs to undergo browning and accompanied mitochondrial activities in response to the stimuli.

### Nuclear receptor ligand screening with CD10 as a biomarker identifies dexamethasone as a promoter and retinoic acid as an inhibitor of adipogenesis

**Fig. 4:**
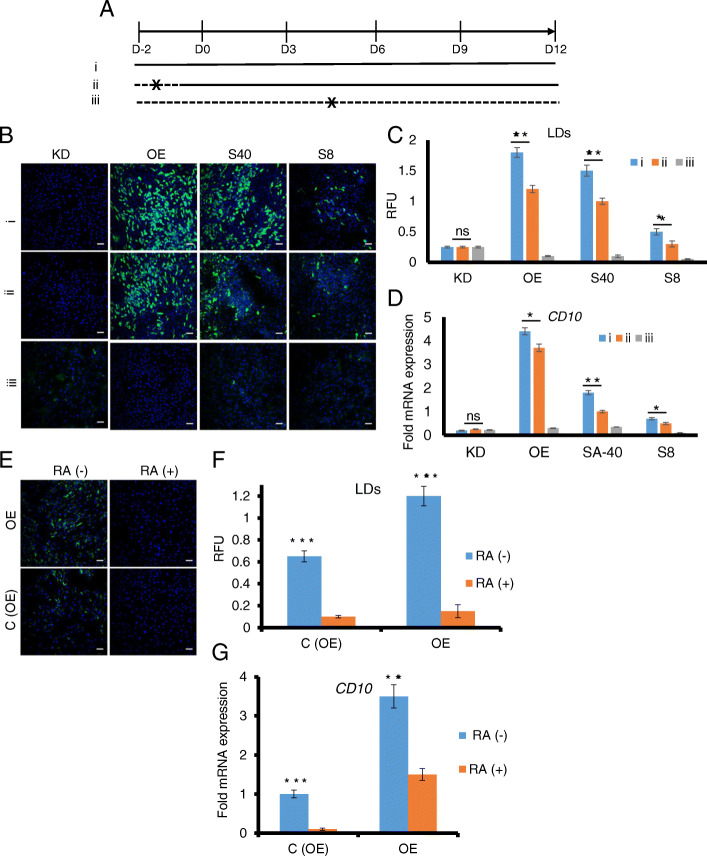
Drug screening with CD10 as a marker identified dexamethasone and retinoic acid, which exhibit stimulatory and inhibitory effects on adipogenesis, respectively. **a** Dexamethasone (10 μM) was added under three conditions such as (i) D-2 to D12 (pre-induction and adipogenic stimulation), (ii) D0 to D12 (only adipogenic stimulation), and (iii) no addition. The presence and absence of dexamethasone are shown as solid and dotted lines, respectively. **b** Representative merged images of AdipoRed staining of lipids (in green) and Hoechst 33342 staining of nuclei (in blue) are shown in CD10 KD, CD10 OE, S40, and S8 ASCs. The scale bar represents 100 μm. **c** The LDs are quantified as mentioned earlier as a read-out of adipogenesis levels. Statistical analysis was performed using Student’s paired *t* test (*n* = 3) between conditions (i) and (ii), ** *p* < 0.01, *p < 0.05, non-significant (ns) represents *p* value > 0.05. **d** Relative mRNA levels of *CD10* are quantified under the indicated conditions of dexamethasone addition normalized to *RPL27*. Fold expression changes are compared to the subject S40 (ii). Pre-treatment with dexamethasone significantly increased *CD10* expression and subsequently lipid accumulation except for the CD10 KD line. Statistical analysis was performed using Student’s paired *t* test (*n* = 3) between condition (i) and (ii), **p < 0.01, *p < 0.05, non-significant (ns) represents *p* value > 0.05. Each value is the mean ± SEM from three independent replicates. **e** Representative merged images of AdipoRed staining of lipids (in green) and Hoechst 33342 staining of nuclei (in blue) are compared between CD10 OE and control ASCs to see the effect of retinoic acid (RA) on adipogenesis. Equivalent amount of either DMSO or RA (10 μM) was added. The scale bar represents 100 μm. **f** LDs was quantified based on MATLAB programming. Addition of RA significantly reduced LDs accumulation. **g** Relative mRNA levels of *CD10* in presence or absence of RA were quantified. Fold expression changes are compared to C (OE) values. RA significantly reduced CD10 expression, and CD10 OE does not rescue RA-mediated adipogenic impairment. Each value is the mean ± SEM from three independent replicates. Significance was assessed by using Student’s paired *t* test: ***p < 0.001

Our results indicate that CD10 can be a useful biomarker for pro-adipogenic capacities in drug screening. As a proof-of-concept, a mini-screening study using high content imaging of ASCs treated with nuclear receptor ligand library (with 76 ligands) was performed as described in the “Materials and methods” section. Nuclear receptor ligands were chosen because many members of this superfamily have been shown to play definitive roles in the processes of adipocyte development [23–25]. The top hit that increased CD10 expression was dexamethasone, one of the standard ingredients of adipogenic cocktail. In contrast, retinoic acid, a metabolite of vitamin A (retinol), showed maximum inhibition (Fig. S4). To study the pro-adipogenic potential of dexamethasone in detail, we divided the adipogenic differentiation processes into 3 schemes (Fig. 4a) and examined them on low (CD10 KD), high (CD10 OE), and intermediate CD10-expressing (S40 and S8) ASCs. In (i), ASCs were pre-treated with dexamethasone for ~ 48 h and maintained throughout the adipogenic process (D-2 to D12); in (ii), dexamethasone was added during the adipogenic treatment without pre-treatment (D0 to D12); and in (iii), no dexamethasone was added at all. Dexamethasone was necessary for adipogenesis to take place as negligible LDs accumulated in the absence of dexamethasone in all the lines including CD10 OE ASCs (Fig. 4b, c). Pre-treatment with dexamethasone also showed a significant increase of *CD10* expression, which in turn resulted in heightened lipid accumulation in all the cell lines except for the CD10 KD cells (Fig. 4b–d). These findings suggest that while CD10 or dexamethasone alone is not sufficient for inducing adipogenesis, CD10 and dexamethasone are important and complementary in enhancing adipogenesis. In the absence of either CD10 or dexamethasone, the adipogenic process is significantly inhibited.

On the other hand, pre-treatment and maintenance with retinoic acid in the differentiation cocktail completely abrogated lipid accumulation and significantly decreased *CD10* expression compared to the DMSO control (Fig. 4e–g), which is in agreement with our previous study [13]. CD10 OE could not relieve the retinoic acid-mediated adipogenic defects. Taken together, our screening using CD10 as a marker could successfully identify drugs that profoundly affect adipogenesis, which further validates CD10 as a prospective marker of mature adipocyte quality.

## Discussion

Studying adipogenesis of ASCs is important for both pathophysiological understanding and quality control aspects of stem cell therapy. It is now evident that adipogenic capability of ASCs depends upon the quality of their originated adipose tissue [6, 12, 26]. There is substantial evidence that imbalance in lipid homeostasis is a major etiological factor resulting in lipo-toxicity and cell death and contributes towards chronic diseases such as insulin resistance and diabetes, cardiovascular disease, immunological and inflammatory disorders, and some types of cancer [27–32]. Therefore, understanding changes in lipid metabolism during stem cell differentiation may offer new insights that could lead to the development of novel, cell-based diagnostic and therapeutic strategies. In general, the ability of ASCs to fully differentiate into mature adipocytes is correlated with the quality of fat tissue that affects metabolic outcomes [6, 9, 13]. Thus, compounds that promote adipogenesis of ASCs may be good candidates for ultimately improving such metabolic diseases. Since ASCs are mesenchymal stem cells that possess trilineage differentiation capacities, the adipogenesis assay is one of the common methods to assess their stem cell potency. However, it typically takes about 2 weeks to perform adipocyte differentiation assay, which may be too time-consuming and laborious for high throughput screening and routine stem cell differentiation protocols. In this paper, we report that CD10 offers a convenient marker that predicts how well ASCs differentiate into mature adipocytes (Fig. 1). Analysis of ASCs with CD10 takes 2 or less days, versus approximately 14 days for the adipocyte differentiation assay. As a proof-of-concept drug screening study, we found that dexamethasone, which significantly induces CD10 expression, enhances adipogenesis substantially (Fig. 4). In contrast, treatment with retinoic acid, which significantly reduces CD10 expression and inhibits adipogenesis, is consistent with our previous report [13]. These results indicate the suitability of CD10 as a prospective marker to investigate the quality of adipocytes with relative ease and speed.

We also found that CD10 directly affects lipid accumulation during adipogenesis (Fig. 1). Stable KD of CD10 reduces whereas its OE increases the accumulation of LDs. Investigation of ASCs from human subjects also confirmed tight correlation between basal CD10 levels and later adipogenesis degrees. These results suggest that it is possible to assess the quality of fat from individuals by examining the levels of intrinsic CD10 as a prospective marker in ASCs. There has been increasing interest to employ ASCs clinically for a number of metabolic disease conditions [8]. In addition to fat tissue for transplantation, ASCs now are actively investigated to improve metabolic outcomes in chronic diseases such as obesity and diabetes [33, 34]. In the future, it may be possible to use CD10 to screen and isolate a population of healthy and competent ASCs that are suitable for transplantation and capable of forming good quality fat tissue that can improve metabolic outcomes.

CD10 is a 100-kDa glycoprotein with neutral endopeptidase activity (NEP) which metabolizes bioactive proteins involved in various physiological processes and regulates their extracellular availability for receptors in the kidney and circulatory and nervous system [35, 36]. CD10 can directly mediate intracellular signaling event via glycosylphosphatidylinositol (GPI) complexes [37]. We found that CD10-dependent lipid accumulation was non-canonical, independent of classical adipogenic pathways that are mediated through the master adipogenic regulator, PPARγ (Fig. S2). It is believed that almost all adipogenic processes, including lipid accumulation, in mature adipocytes are mediated by PPARγ [38, 39]. Natriuretic peptides (NP) and major hormones stimulating catabolic pathway in humans,and have been reported to be the direct substrates of CD10 [29, 40, 41]. In adipocytes, atrial NP (ANP) and brain type NP (BNP) stimulated NP receptor A and induce a cGMP dependent pathway [42, 43]. Increased intracellular cGMP levels activate cGMP-dependent protein kinase G, which in turn, induces lipolysis through phosphorylation of HSL [42]. After phosphorylation, HSL is translocated into the LDs and acts together with ATGL, accelerating the lipolytic process (Fig. S5). Together, ATGL and HSL are responsible for about 95% of TAG hydrolysis [43–47]. We observed upregulation of both HSL and ATGL activities in CD10 KD cells, implying that CD10 mediates suppression of endogenous lipolysis, and in turn, promotes lipid accumulation in mature ASCs (Fig. 3 and Fig. S3, S5). This CD10-induced inhibition of lipolysis may act as an adaptive protective mechanism to minimize FA release and its deleterious consequences on systemic metabolism in obese and insulin-resistant subjects [48–50]. Previously, the lipolytic process was believed to depend primarily upon PPARγ-mediated pathways in mature adipocytes [51–53]. In contrast, our current finding demonstrates a heretofore unrecognized role of a PPARγ-independent factor, CD10, to be involved in lipid accumulation and maturation of adipocytes (Fig. S5).

The inducible browning (beige) adipocytes differ in origin and possess a distinct molecular signature from classical brown adipocytes; however, they both share similar characteristics such as high mitochondria content and UCP1 expression upon activation [54]. Several studies have shown that the same stimuli that increased classical brown fat activity in mice also resulted induced browning [55–58]. These studies suggest that they may be reproducible in man and indicate that inducing or transplanting brown/beige fat may be potential strategies for treating obesity and its associated disorders. In this study, we have found that basal CD10 level is correlated with the browning degree of ASCs, establishing CD10 as the prospective marker to indicate browning capability of stem cells (Fig. 2). It is presumed that the higher lipid accumulation found in CD10-high mature ASCs also is a prerequisite for enhanced browning capability of these cells, which upon appropriate stimulation leads to higher mitochondrial oxidative respiration during browning (Fig. 3H).

## Conclusions

Our data demonstrate that CD10 regulates a novel, non-canonical adipocyte maturation process, which is essential for ASC maturation into high-quality WAT and browning. Thus, CD10 expression in ASCs may play an important role in preventing the development of obesity and other metabolic complications. Moreover, as a functional biomarker, CD10 promises to have clinical relevance and diverse applications for drug screening, differentiation assessment, and therapeutics related to obesity and diabetes.

## Supplementary Information

**Additional file 1.**

## Data Availability

The datasets analyzed and materials used during the current study are available either commercially or from the corresponding author upon request.

## Abbreviations

ASCs: Adipose-derived stem cells
PPARγ: Peroxisome proliferator-activated receptor gamma
WAT: White adipose tissue
BAT: Brown adipose tissue
UCP1: Uncoupling protein-1
LDs: Lipid droplets
HSL: Hormone-sensitive lipase
ATGL: Adipose triglyceride lipase
TGs: Triglycerides
FAs: Free fatty acids
NEP: Neutral endopeptides
NP: Natriuretic peptides
ANP: Atrial NP
BNP: Brain type NP
NPRA: NP guanyly cyclase receptor of the A subtype
FSK: Forskolin
