# Supplementary material for: CD10 marks non-canonical PPARγ-independent adipocyte maturation and browning potential of adipose-derived stem cells
Source: Stem Cell Res Ther. 2021 Feb 4;12:109. doi: 10.1186/s13287-021-02179-y (PMC7863460; doi:10.1186/s13287-021-02179-y)
Supplement: Supplementary file 1 — Additional file 1. [file 13287_2021_2179_MOESM1_ESM.zip › CD10 SI Final.docx]

**Supplementary Figures and Tables**

A


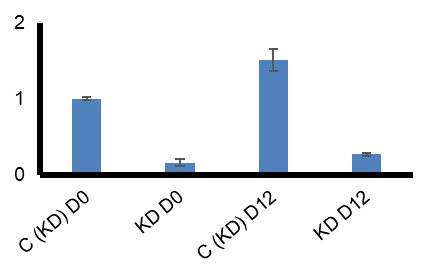


***

**

S27

Fold mRNA expression

C


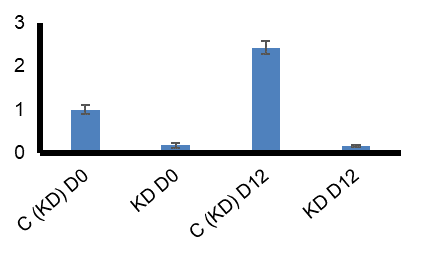


**

***

S23

Fold mRNA expression


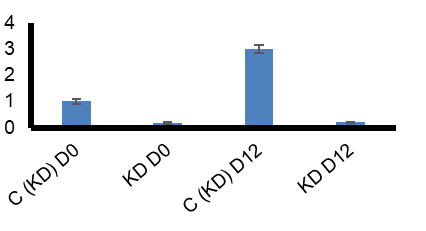


***

**

S13

Fold mRNA expression

B


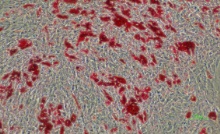

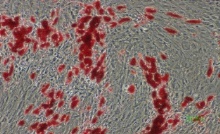

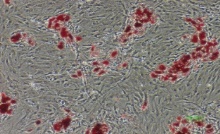


S13 S23 S27


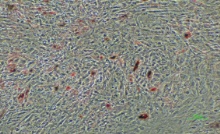

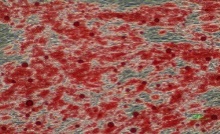

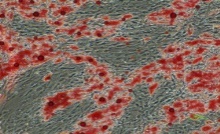


C (KD)

KD

D

E


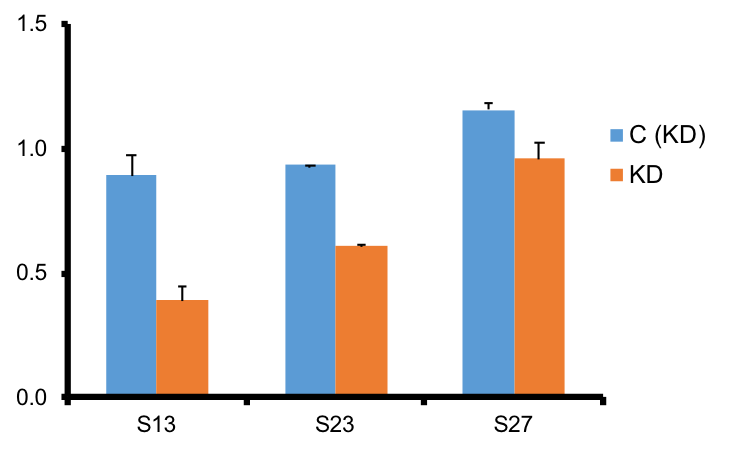


***

*

**

Absorbance (500nm)

S13 S23 S27

KD

C (KD)

Fig. S1

**Fig. S1.** CD10 determines adipogenic maturation and browning capacities of ASCs. Relative mRNA levels of CD10 of **(A)** S13 **(B)** S23 and **(C)** S27 are quantified by qRT-PCR normalized to RPL27. Fold expression changes are compared to control cells at D0 for each subject. Each value is the mean ± SEM from three independent replicates. **(D)** Representative brightfield images of Oil Red O staining (in red) of CD10 KD and control ASCs are shown. The scale bar represents 100 µm. **(E)** Quantification of Oil Red O staining is shown. Reduced lipid accumulation in CD10 KD ASCs is observed compared to the controls in all 3 subjects. Each value is the mean ± SEM from three independent replicates.

A


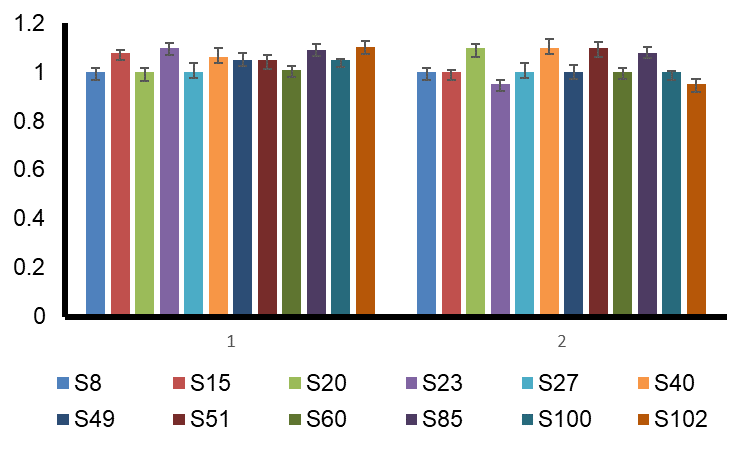


ns

Fold mRNA expression

ns

*PPARG*

*FABP4*

B

C


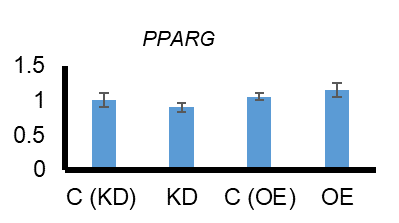


ns

ns

Fold mRNA expression

B

C


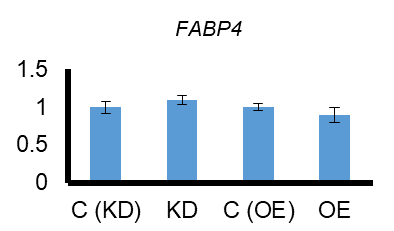


ns

ns

Fold mRNA expression

C (OE)


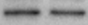

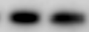

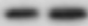


C (KD)

PPARG

KD

OE

GAPDH


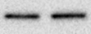

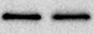


pAKT


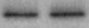

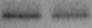


AKT


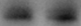

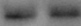

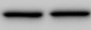

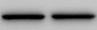


GAPDH

pp70S6

p70S6


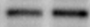


GAPDH


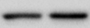

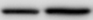

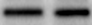

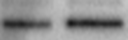

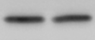

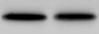

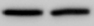

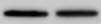

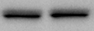

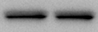

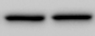

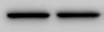


ATF4

GAPDH

ATF6

GAPDH

C (KD)

KD

OE

C (OE)

C (KD)

C (OE)

D


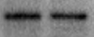

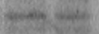


pAMPK

KD

OE


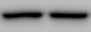

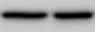


GAPDH


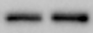

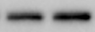


AMPK


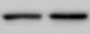

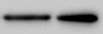


GAPDH


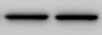

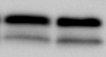

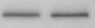

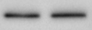

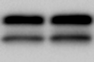


p62

LC3


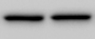


GAPDH

Fig. S2

**Fig. S2.** **CD10 functions non-canonically independent of classical adipogenic pathways**. **(A)** Relative mRNA levels of *PPARG and FABP4* quantified by qRT-PCR normalized to *RPL27* are shown for differentiated ASCs from 12 subjects. Fold expression changes are compared to S8. ns denotes non-significant, p >0.05. Relative mRNA levels of **(B)** *PPARG and* **(C)** *FABP4* are displayed for differentiated CD10 KD, OE and control cells from qRT-PCR analysis normalized to *RPL27*. ns denotes non-significant. **(D)** Western blot analysis shows expression of PPARG, phospho-AKT (P-S473), total AKT, phospho-p70S6 (P-T470, P-T389), p70S6 (P-T470), phospho-AMPK (P-T172), total AMPK, p62, LC3, ATF4 and ATF6 proteins in CD10 KD and OE cells compared to the controls. GAPDH was used as internal control.

A

B

NEFA content (mg)

/mg protein


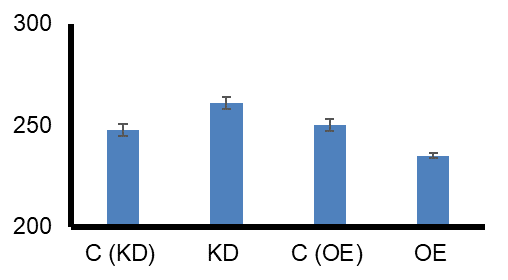


ns

ns

Triglyceride content (mg)

/mg protein


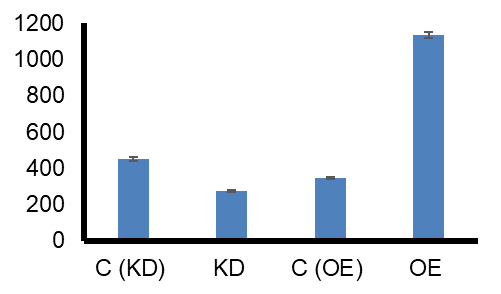


*

***

C

D

Glycerol release (mg)

/mg protein


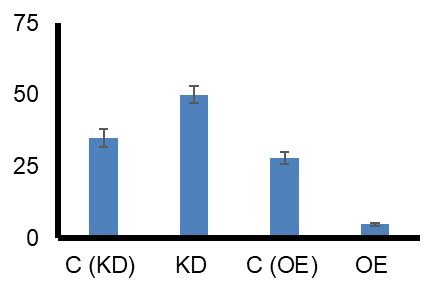


**

*

NEFA release (mg)

/mg protein


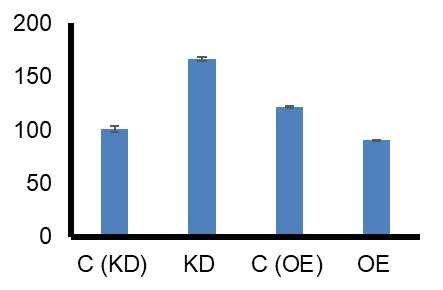


***

***

Fig. S3

**Fig. S3.** **CD10 exerts reduced hydrolysis of lipid TGs into FAs and Glycerol. (A)** TGs content of mature CD10 KD, OE and control cells are shown normalized to the total protein levels. **(B)** Intracellular and **(C)** Secreted levels of non-esterified FAs (NEFAs) are shown normalized to the total protein levels. **(D)** The amount of glycerol released into the medium are quantified from CD10 KD, OE and control cells normalized to intracellular protein values. All statistical significance was assessed by using Student’s paired t-test, ***p <0.001, **p <0.01, *p, <0.05, ns; non-significant, p>0.05. All the measurements are taken from each of six replicate wells.


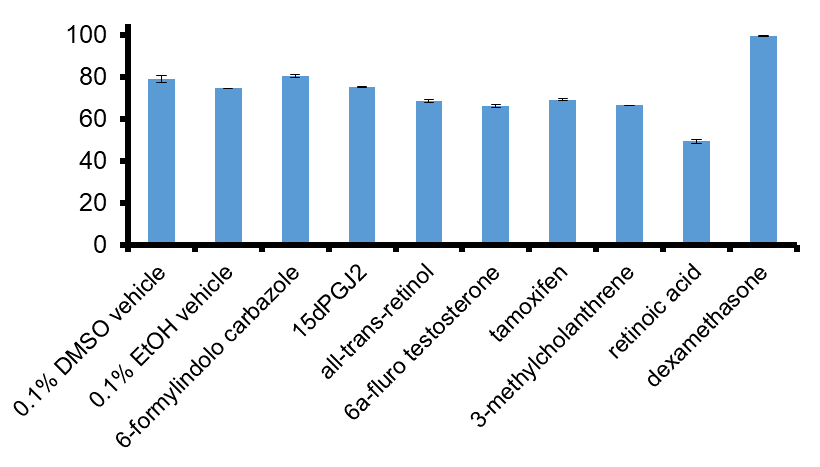


Percentage of CD10^+^cells

A

B


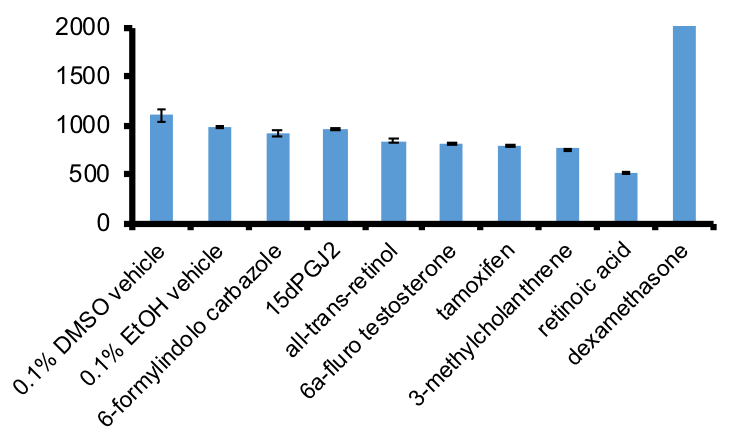

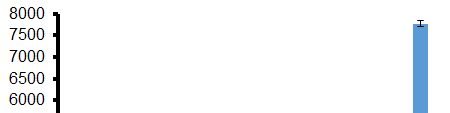


Average CD10-associated fluorescence intensity

Fig. S4

**Figure S4. Effects of selected hits from nuclear receptor ligand library on expression of CD10 in ASCs. (A)** Percentage of CD10 positive cells and **(B)** Average CD10-FITC intensity are assessed by flow cytometry analyses. Each value is the mean ± SEM from 3 separate wells of cells cultured under the same specified condition. Nuclear receptor ligand library screening identifies dexamethasone as a stimulator and retinoic acid as an inhibitor of CD10 expression.

Fig. S5


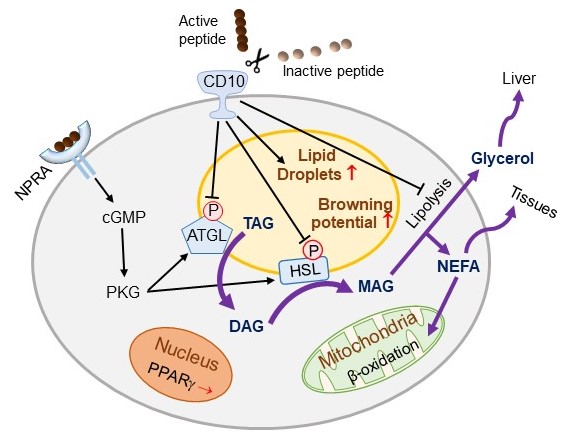


**Figure S5. CD10 exerts a direct role in adipocyte maturation of ASCs by regulating intracellular lipolysis.** CD10 is known to inactivate biologically active peptides (ANPs) by hydrolyzing the N-terminal hydrophobic amino acids residues. In absence of CD10, ANP might build up and bind to NP guanyly cyclase receptor of the A subtype (NPR-A), resulting in the sustained increase of intracellular cGMP. Activated cGMP leads to the phosphorylation of lipases such as HSL and ATGL at the LD surface. Phosphorylated HSL and ATGL increases the breakdown of TAG stored in adipocytes to NEFA and glycerol, leading to mitochondrial respiration / β-oxidation. CD10-mediated inactivation of HSL and ATGL results in deactivation of lipolysis, resulting in increased LDs accumulation during adipogenesis without affecting the PPARγ-dependent classical adipogenic pathway.

**Table S1:** Donor information

| **Subject** | **Age** | **Sex** | **T2DM/ NDM** |
| --- | --- | --- | --- |
| S8 | 40-50 | F | T2DM |
| S13 | 30-40 | F | T2DM |
| S15 | 20-30 | F | T2DM |
| S20 | 20-30 | F | T2DM |
| S23 | 30-40 | M | NDM |
| S27 | 20-30 | F | NDM |
| S29 | 30-40 | F | NDM |
| S40 | 50-60 | F | NDM |
| S49 | 50-60 | F | NDM |
| S51 | 30-40 | F | NDM |
| S60 | 20-30 | F | NDM |
| S85 | 50-60 | F | NDM |
| S100 | 50-60 | F | T2DM |
| S102 | 50-60 | F | NDM |

**Table S2:** Primers used in this study

| **Gene** | **Forward** | **Reverse** |
| --- | --- | --- |
| RT RPL27 | GGGTGGTTGCTGCCGAAATG | TGGCTGTAGGGGCGATCTGA |
| RT UCP1 | ACGGGTCTTTGGAAAGGGACTA | GCGATAAGAGCCGACACCAA |
| RT CD10 | GTCTTCCCAGCCGGCATTCT | AGTTTCTGCCATTGTCATCGAAGC |
| RT PPARG | GACAGGAAAGACAACAGACA AATC | GGGGTGATGTGTTTGAACTTG |
| RT FAPB4 | CCTTTAAAAATACTGAGATTT CCTTCA | GGACACCCCCATCTAAGGTT |
| iRFP720 | ACAAGCTAGCGCCACCATGGCGGAAGGATCCGTC | ACAAGCGGCCGCTCAAGGTCCAGGGTTCTCCTCCACGTCTCCAGCCTGCTTCAGCAGGCTGAAGTTAGTAGCTCCGCTTCCCTCTTCCATCACGCCGATCTG |
| CD10 | ACAAGCTAGCACAAGGGTCGACGCCACCATGGCAAGTCAGAAAGT | ACAAGCGGCCGCTCACCAAACCCGGCACTTC |
| iRFP720-P2A | ACAAGCTAGCGCCACCATGGCGGAAGGATCCGTC | ACAAGCGGCCGCAGGTCCAGGGTTCTCCTCCACGTCTCCAGCCTGCTTCAGCAGGCTGAAGTTAGTAGCTCCGCTTCCCTCTTCCATCACGCCGATCTG |
| CD10 shRNA | *GATCCA***GAACAGTAGGTGACACTAT**ACTCGAGA**ATAGTGTCACCTACTGTTC***TTTTTTCCAAG* | |

**Table S3:** Antibodies used in this study

| **Antibodies** | **Source** | **Identifier** |
| --- | --- | --- |
| Mouse monoclonal CD10 (Clone 56C6) | Leica Microsystmes | Cat# CD10-270-L-CE |
| phospho-AKT (S473) | Cell Signaling Technology | 4058S |
| AKT | Cell Signaling Technology | 9272S |
| phospho-p70S6 (T389) | Cell Signaling Technology | 9205S |
| p70S6 | Cell Signaling Technology | 9202S |
| phospho-AMPK (T172) | Cell Signaling Technology | 2535S |
| AMPK | Cell Signaling Technology | 5831S |
| p62 | Cell Signaling Technology | 5114S |
| LC3 | Cell Signaling Technology | 2775S |
| ATF4 | Cell Signaling Technology | 11815S |
| ATF6 | Novus Biologicals | NBP1-40256 |
| COXIV | Cell Signaling Technology | 4850P |
| phospho-HSL (S660) | Cell Signaling Technology | 4126S |
| HSL | Cell Signaling Technology | 4107S |
| phospho-ATGL (S406) | Abcam | ab135093 |
| ATGL | Abcam | ab99532 |
| GAPDH | Cell Signaling Technology | 2118L |
