# Supplementary material for: CD10 marks non-canonical PPARγ-independent adipocyte maturation and browning potential of adipose-derived stem cells
Source: Stem Cell Res Ther. 2021 Feb 4;12:109. doi: 10.1186/s13287-021-02179-y (PMC7863460; doi:10.1186/s13287-021-02179-y)
Supplement: Supplementary file 1 — Additional file 1. [file 13287_2021_2179_MOESM1_ESM.zip › Table S4 Final.pdf]

Table S4: First screening results of nuclear receptor ligand library using CD10 as a marker with flow cytometry analysis and ImageEpress analysis.

	Flow Cytometry Analysis							ImageXpress Analysis					
Ligand	Well Location	P2 #Events	P2 %Parent	P2 FITC-A Mean	% Fluor Intensity relative to Control (A2&A3)	P3 #Events	P3 %Parent	Fluor 1	Fluor 2	Fluor 3	Fluor 4	Mean Fluor Intensity	% Fluor Intensity relative to Control (A2&A3)
(empty)	A1	306	89	108	81.8	0	0.0	2.49	8.82	5.47	6.22	5.8	27.6
DMSO (control)	A2	462	97.5	132	**100.0**	9	1.9	20	26.82	22.88	20.44	**22.5**	**108.0**
Ethanol (control)	A3	141	100	132	**100.0**	2	1.4	17.29	20.88	13.21	25.37	**19.2**	**92.0**
25-Hydroxyvitamin D3	B1	95	82.6	167	126.5	5	5.3	18.07	24.28	29.47	17.72	22.4	107.3
Retinoic acid, all trans	B2	167	77.7	144	109.1	7	4.2	19.19	9.63	8.91	32.9	**17.7**	**84.6**
9-cis Retinoic acid	B3	148	80	135	102.3	6	4.1	16.66	23.52		28.79	23.0	110.2
13-cis Retinoic acid	B4	296	78.9	125	94.7	4	1.4	18.46	19.11	14.65	14.94	16.8	80.5
4-Hydroxyphenylretinamide	B5	372	58.7	152	115.2	19	5.1	30.91	29.38	9.81	22.11	23.1	110.5
AM-580	B6	442	79.2	140	106.1	14	3.2	17.79	15.81	16.44	13.86	16.0	76.6
TTNPB	B7	366	79.6	128	97.0	11	3.0	18.25	12.86	20.33	18	17.4	83.2
Methoprene acid	B8	941	80.4	139	105.3	29	3.1	24.2	25.24	27.9	25.64	25.7	123.4
WY-14643	B9	574	74.1	143	108.3	29	5.1	20.37	18.08	23.46	21.22	20.8	99.6
Ciglitazone	B10	481	78.2	141	106.8	14	2.9	25.18	23.77	15.44	22.53	21.7	104.2
Tetradecylthioacetic acid	B11	862	75.2	137	103.8	38	4.4	18.54	15.77	17.89	16.96	17.3	82.9
5,8,11,14-Eicosatetraynoic acid	B12	383	68.3	168	127.3	30	7.8	15.13	15.71	100.77	11.11	35.7	171.0
6-Formylindolo [3,2-B] carbazole	C1	735	66.5	443	**335.6**	486	**66.1**	73.16	66.87	56.74	64.49	**65.3**	**313.1**
Diindolylmethane	C2	441	80.3	135	102.3	7	1.6	17.42	18.41	21.64	20.82	19.6	93.8
Acetyl-S-farnesyl-L-cysteine	C3	325	80.2	134	101.5	12	3.7	21.21	14.56	16.97	23.67	19.1	91.6
S-Farnesyl-L-cysteine methyl ester	C4	694	79.6	155	117.4	53	7.6	32.26	33.37	41.64	35.31	35.6	170.9
N-Acetyl-S-geranygeranyl-L-cysteine	C5	825	78.6	144	109.1	36	4.4	23.75	28.52	36.27	33	30.4	145.7
AGC (Acetyl-geranyl-cysteine)	C6	681	80	118	89.4	17	2.5	17	17.61	12.84	12.6	15.0	72.0
Farnesylthioacetic acid	C7	398	82.4	120	90.9	8	2.0	12.73	15.04	10.51	20.45	14.7	70.4
Bezafibrate	C8	1061	77.9	142	107.6	47	4.4	18.55	20.23	21.02	26.97	21.7	104.0
LY 171883	C9	382	74	152	115.2	21	5.5	18.47	21.15	17.49	17.29	18.6	89.2
15-Deoxy-D12,14-prostaglandin J2	C10	275	59.1	179	**135.6**	34	**12.4**	17.06	22.64	17.74	20	19.4	92.8
Troglitazone	C11	630	73.8	138	104.5	25	4.0	15.25	17.91	12.65	19.16	16.2	77.9
CITCO	C12	471	80.2	139	105.3	15	3.2	13.97	20.89	17.44	12.02	16.1	77.1
Paxilline	D1	801	84.9	149	112.9	39	4.9	15.04	26.6	28.07	25.31	23.8	113.9
24(S)-Hydroxycholesterol	D2	7	58.3	119	90.2	0	0.0	19.66	23.64	23.23	29.41	24.0	115.0
24(S),25-Epoxycholesterol	D3	127	81.4	130	98.5	1	0.8	21.15	37.87	23.72	30.51	28.3	135.7
Pregnenolone-16(alpha)-carbonitrile	D4	1185	79.9	142	107.6	56	4.7	23.12	24.31	28.29	25.6	25.3	121.4
Carbacyclin	D5	512	83.5	152	115.2	26	5.1	29.27	32.82	35.88	29.47	31.9	152.7
Clofibric acid	D6	1079	79.5	130	98.5	36	3.3	21.18	21.41	34.62	22.5	24.9	119.5
BADGE	D7	370	81.9	121	91.7	8	2.2	13.41	11.5	4.35	9.24	9.6	46.1
GW 9662	D8	997	74.8	140	106.1	52	5.2	21.1	18.37	18.17	22.27	20.0	95.8
Gemfibrozil	D9	833	80.6	136	103.0	34	4.1	21.9	14.51	16.92	22.06	18.8	90.3
GW 7647	D10	834	82.3	136	103.0	18	2.2	17.96	14.97	12.7	19.98	16.4	78.6
3,5-Diiodo-L-thyronine	D11	1003	80	147	111.4	52	5.2	22.21	21.87	20.09	12.27	19.1	91.6
3,5-Diiodo-L-tyrosine	D12	411	76	148	112.1	24	5.8	28.95	19.11	36.95	13.52	24.6	118.1
all-trans-Retinol	E1	152	74.1	322	**243.9**	72	**47.4**	41.95	41.91	24.25	31.63	**34.9**	**167.5**
13-cis-Retinol	E2	699	84	139	105.3	32	4.6	24.39	21.3	22.99	23.75	23.1	110.8
Retinyl acetate	E3	790	81.6	135	102.3	20	2.5	16.58	16.02	16.84	18.01	16.9	80.8
3,5-Diiodo-4-hydroxyphenylpropionic acid	E4	1036	78.5	132	100.0	30	2.9	21.45	21.04	26.08	21.93	22.6	108.5
Cholic acid	E5	1061	83.2	136	103.0	47	4.4	21.52	23.59	21.26	26.44	23.2	111.2
Deoxycholic acid	E6	1148	80.4	141	106.8	63	5.5	23.91	22.38	27.12	22.91	24.1	115.4
Chenodeoxycholic acid	E7	1402	81.8	130	98.5	39	2.8	23.59	27.52	22.81	23.22	24.3	116.4
Glycocholic acid	E8	1174	78.7	136	103.0	30	2.6	25.67	21.93	24.05	20.12	22.9	110.0
Glycodeoxycholic acid	E9	1068	77.9	134	101.5	26	2.4	25.18	24.69	18.31	14.93	20.8	99.6
Taurocholic acid	E10	981	80.5	129	97.7	16	1.6	16.14	16.69	18.54	22.27	18.4	88.2
Taurodeoxycholic acid	E11	954	78.9	137	103.8	38	4.0	16.31	16.41	24.32	21.75	19.7	94.4
Rifampicin	E12	413	78.8	136	103.0	14	3.4	13.01	11.15	15.86	21.76	15.4	74.0
Dexamethasone	F1	157	56.9	578	**437.9**	114	**72.6**	65.27	59.53	67.78	61.69	**63.6**	**304.7**
Lithocholic acid	F2	752	80.8	139	105.3	38	5.1	28.02	23.66	23.22	26.51	25.4	121.5
5b-Pregnan-3,20-dione	F3	1171	80.9	148	112.1	71	6.1	17.53	24.96	23.36	25.76	22.9	109.8
Adapalene	F4	270	87.9	148	112.1	10	3.7	12.49	18.47	11.18	11.84	13.5	64.7
Farnesol	F5	964	84.7	140	106.1	33	3.4	22.08	21.98	17.33	26.5	22.0	105.3
3a, 5a-Androstenol	F6	1237	80.5	145	109.8	65	5.3	17.06	21.78	16.18	17.81	18.2	87.3
3α, 5α-Androstanol	F7	889	82.1	131	99.2	27	3.0	24.18	16.08	19.85	16.53	19.2	91.8
Z-Guggulsterone	F8	1036	80.7	143	108.3	51	4.9	24.71	19.76	27.06	23.4	23.7	113.8
TCPOBOP	F9	1042	78.1	141	106.8	58	5.6	23.37	24.5	23.06	23.2	23.5	112.8
N-Oleoylethanolamide	F10	764	82.6	124	93.9	17	2.2	18.57	16.53	10.72	15.7	15.4	73.7
GW4064	F11	679	79.4	147	111.4	32	4.7	16.66	22.1	19.1	13.16	17.8	85.1
Geranylgeraniol	F12	326	76.9	136	103.0	4	1.2	13.23	10.54	16	17.85	14.4	69.1
6a-Fluorotestosterone	G1	55	56.7	350	**265.2**	33	**60.0**	59.64	49.72		52.55	**54.0**	**258.7**
Tamoxifen	G2	381	89.2	178	**134.8**	30	7.9	32.14	19.96	22.31	27.72	25.5	122.4
Mifepristone	G3	650	80.5	153	115.9	39	6.0	24.31	30.46	26.74	23.25	26.2	125.5
Estrone	G4	932	78.7	125	94.7	22	2.4	16.97	19.64		19.22	18.6	89.2
13(S)-Hydroxy-9Z,11E-octadecadienoic acid	G5	657	82.2	132	100.0	14	2.1	22.66	26.68	24.51	16.28	22.5	108.0
Cortisone	G6	1175	81.1	136	103.0	40	3.4	23.68	25.7	20.8	29.91	25.0	119.9
Progesterone	G7	749	85.3	156	118.2	51	6.8	22.16	24.25	25.2	30.19	25.5	122.0
17b-Estradiol	G8	881	80.9	127	96.2	25	2.8	17.18	13.61	17.98	17.51	16.6	79.4
Pregnenolone	G9	867	81	137	103.8	36	4.2	24.26	26.82	19.41	17.54	22.0	105.5
Androstenedione	G10	887	80.5	152	115.2	45	5.1	26.71	20.63	19.45	16.11	20.7	99.3
1a,25-Dihydroxyvitamin D3	G11	151	87.8	151	114.4	4	2.6	13.08	16.02	8.54	6.76	11.1	53.2
Docosa-4Z,7Z,10Z,13Z,16Z,19Z-hexaenoic acid	G12	287	74.2	148	112.1	10	3.5	19.41	14.09	27.72	13.64	18.7	89.7
3-Methylcholanthrene	H1	74	75.5	221	**167.4**	13	**17.6**	22.23	21.21		21.49	21.6	103.7
Acitretin	H2	106	79.7	131	99.2	4	3.8	11.95	19.67	39.7	24.53	24.0	114.9
Pioglitazone	H3	321	84.9	155	117.4	26	8.1	12.06	15.43	22.95	19.81	17.6	84.2
4-Hydroxyretinoic acid	H4	185	85.6	129	97.7	5	2.7	6.09	9.79	6.06	32.85	13.7	65.7
